# Risk factors associated with high-dose methotrexate induced toxicities in primary central nervous system lymphoma

**DOI:** 10.3389/fphar.2025.1561818

**Published:** 2025-08-04

**Authors:** Wenshu Li, Sitian Zhang, Ruoyun Wu, Ying Li, Shifeng Wei, Lin Fu, Xuefei Sun, Yuanbo Liu, Zhigang Zhao, Shenghui Mei

**Affiliations:** ^1^ Department of Pharmacy, Beijing Tiantan Hospital, Capital Medical University, Beijing, China; ^2^ Department of Clinical Pharmacology, College of Pharmaceutical Sciences, Capital Medical University, Beijing, China; ^3^ School of Basic Medical Sciences, Capital Medical University, Beijing, China; ^4^ Department of Hematology, Beijing Tiantan Hospital, Capital Medical University, Beijing, China

**Keywords:** high dose methotrexate, nephrotoxicity, hepatotoxicity, hematotoxicity, risk factors, single nucleotide polymorphism

## Abstract

High-dose methotrexate (HDMTX) is the cornerstone of the treatment for primary central nervous system lymphoma (PCNSL). The prevention of drug-induced toxicities is critical. This study aims to identify key factors associated with HDMTX-induced toxicities (hematotoxicity, hepatotoxicity and nephrotoxicit) in 713 Chinese PCNSL patients undergoing 3021 HDMTX treatment courses. Demographic data, administration information, laboratory tests, area under the curve, co-medications, and 30 single nucleotide polymorphisms were collected to analyze the association of HDMTX-related toxicities using PLINK and SPSS. Higher ALB level, female, ABCB1 rs1045642, MTHFR rs1801131, and MTHFD1 rs2236225 were associated with lower risk of anemia, while the combination of furosemide, torasemide, bumetanide, and levetiracetam associating with higher risk. Co-use of torasemide had higher incidence of neutropenia. Higher level of ALB was correlated with less leukopenia; torasemide and rs2236225 were related to more leukopenia. Female, furosemide, rs1801133, ABCG2 rs2231142, ABCC2 rs717620 were related to more thrombocytopenia, while rs1045642 and high ALB were related to less. Rs1801131 and female were correlated with more hepatotoxicity, whereas furosemide was correlated with less. In nephrotoxicity, female and rs1801394 were correlated with less, MTHFR rs1801131 and rs1801133 were correlated with more. In conclusion, higher ALB levels had a lower risk of HDMTX toxicities; loop diuretics and levetiracetam generally accelerated the occurrence of toxicities. Rs1801133 GG, rs1128503 GG + AG, rs2231142 AA+ AC, rs717620 TT + GT were associated with increased risk of toxicity; rs1045642 TT and rs1801394 GG + AG were less likely to develop toxicity.

## 1 Introduction

Primary central nervous system lymphoma (PCNSL) is a rare and highly aggressive extranodal non-Hodgkin’s lymphoma with a poor prognosis (Flosperg et al., 2024). Methotrexate (MTX), a folic acid antagonist, is widely used to treat various malignancies. High-dose methotrexate (HDMTX), defined as a dose exceeding 500 mg/m^2^, is the cornerstone of PCNSL treatment. Chemotherapy regimens containing HDMTX yield higher survival rates and longer survival times compared to radiotherapy alone or other treatments (Koźmiński et al., 2020; Kim et al., 2012).

Methotrexate inhibits dihydrofolate reductase, disrupting key enzymes involved in DNA and RNA synthesis, thereby preventing the proliferation of rapidly dividing tumor cells (Maksimovic et al., 2020). Methotrexate exhibits non-linear pharmacokinetics, meaning that drug concentration does not proportionally increase with dosage, and its metabolism, transport, and excretion processes are complex. HDMTX is primarily administered intravenously and widely distributed in tissues, with high concentrations in the kidneys, liver, skin, and red blood cells (Mei et al., 2018a; Fischer et al., 2017). MTX has a 50% protein binding rate, so plasma protein levels can affect its pharmacokinetic parameters (Li et al., 2024). Approximately 80%–90% of MTX is excreted unchanged via the kidneys, with the remaining 10% excreted through bile (Methotrexate-methotrexate injection, 2024).

In PCNSL patients, HDMTX pharmacokinetics exhibit significant interindividual variability, mainly in apparent volume of distribution and clearance rate, influenced by factors such as patient pathophysiological status, administration regimen, and polymorphisms in transporters and metabolic enzymes (Mei et al., 2018a). HDMTX treatment can cause various systemic adverse effects, including nephrotoxicity (acute kidney injury in 2%–12% of patients), hepatotoxicity, myelosuppression, and neurotoxicity. Due to these potential toxicities, HDMTX management requires rigorous monitoring and personalized dosing strategies.

This study aims to identify clinical factors associated with HDMTX-induced toxicities and assess the impact of genetic polymorphisms in MTX metabolism pathway and transporters on toxicity occurrence. Early identification of these risk factors can guide clinicians in optimizing dosing strategies, helping to minimize adverse effects while maximizing therapeutic efficacy.

## 2 Methods

### 2.1 Patient enrolment and treatment

This study received approval from the Ethics Committee of Beijing Tiantan Hospital, Capital Medical University, Beijing, P.R. China (KY 2019-072-02) and was performed consistent with the guidelines of the Declaration of Helsinki. Data were collected through routine clinical monitoring and leftover blood samples. All patients were diagnosed with PCNSL and treated with intravenous HDMTX at Beijing Tiantan Hospital from September 2016 to February 2024. Urine alkalinization with sodium bicarbonate and intravenous hydration (sodium chloride, 1500 mL/m^2^) were administered. Calcium leucovorin regiment was adjusted according to the plasma MTX level (Methotrexate-methotrexate injection, 2024). To control inflammatory side effects, 5–10 mg dexamethasone was intravenously administered before MTX infusion (Cook et al., 2016).

Inclusion criteria: age ≥18 years; no pregnancy or HIV infection; no missing of required information.

### 2.2 Data collection

Clinical data of enrolled patients were retrospectively collected. These data included age, gender, body weight, height, MTX dosing time and dosage/m^2^, C_MTX_, 0–24 h area under curve (AUC_0–24h_), and AUC_0–48h_, albumin (ALB), white blood cell count (WBC), hemoglobin (HGB), platelet (PLT) count, alanine transferase (ALT), aspartate transferase (AST), creatinine (Cr), co-medications, and selected single nucleotide polymorphisms (SNPs).

### 2.3 Toxicity evaluation and plasma MTX concentration

Hematotoxicity (including anemia, neutropenia, leukopenia, and thrombocytopenia), hepatotoxicity and nephrotoxicity were assessed according to the Common Terminology Criteria for Adverse Events (version 5.0) (CTCAE, 2024). Hepatotoxicity and nephrotoxicity were evaluated using the highest values of ALT, AST, and Cr levels during the courses. Grades 1 to 4 of nephrotoxicity are graded 1–1.5 times, 1.5–3 times, 3–6 times, and more than 6 times the upper limit of normal (ULN) respectively. A detailed description of the toxicity assessment criteria was shown in Table 1. Plasma MTX concentrations were determined by a validated ultra-performance liquid chromatography-tandem mass spectrometry method (Mei et al., 2018b).

**TABLE 1:** Toxicity assessment criteria according to common terminology criteria for adverse events (version 5.0).

CTCAE Term	Grade 1	Grade 2	Grade 3	Grade 4	Grade 5
Alanine aminotransferase increased	>ULN - 3.0 x ULN if baseline was normal; 1.5–3.0 x baseline if baseline was abnormal	>3.0–5.0 x ULN if baseline was normal; >3.0–5.0 x baseline if baseline was abnormal	>5.0–20.0 x ULN if baseline was normal; >5.0–20.0 x baseline if baseline was abnormal	>20.0 x ULN if baseline was normal; >20.0 x baseline if baseline was abnormal	-
Aspartate aminotransferase increased	>ULN - 3.0 x ULN if baseline was normal; 1.5–3.0 x baseline if baseline was abnormal	>3.0–5.0 x ULN if baseline was normal; >3.0–5.0 x baseline if baseline was abnormal	>5.0–20.0 x ULN if baseline was normal; >5.0–20.0 x baseline if baseline was abnormal	>20.0 x ULN if baseline was normal; >20.0 x baseline if baseline was abnormal	-
White blood cell decreased	<LLN - 3,000/mm3; <LLN - 3.0 × 10e9/L	<3,000–2000/mm3; <3.0–2.0 × 10e9/L	<2000–1000/mm3; <2.0–1.0 × 10e9/L	<1000/mm3; <1.0 × 10e9/L	-
Neutrophil count decreased	<LLN - 1500/mm3; <LLN - 1.5 × 10e9/L	<1500–1000/mm3; <1.5–1.0 × 10e9/L	<1000–500/mm3; <1.0–0.5 × 10e9/L	<500/mm3; <0.5 × 10e9/L	-
Platelet count decreased	<LLN - 75,000/mm3; <LLN - 75.0 × 10e9/L	<75,000–50,000/mm3; <75.0–50.0 × 10e9/L	<50,000–25,000/mm3; <50.0–25.0 × 10e9/L	<25,000/mm3; <25.0 × 10e9/L	-
Anemia	Hemoglobin (Hgb) <LLN - 10.0 g/dL; <LLN - 6.2 mmol/L; <LLN - 100 g/L	Hgb <10.0–8.0 g/dL; <6.2–4.9 mmol/L; <100–80 g/L	Hgb <8.0 g/dL; <4.9 mmol/L; <80 g/L; transfusion indicated	Life-threatening consequences; urgent intervention indicated	Death

### 2.4 Genotype identification

Thirty SNPs were selected according to the following criteria: with significant influence on MTX pharmacokinetics and/or toxicities; with acceptable mutation frequency (>0.05) in the Chinese population; can be successfully identified by MassArray method (Sequenom, USA). DNA was isolated from the residual monitoring samples, and the genotype of 30 selected SNPs was identified by MassArray method (Ellis and Ong, 2017; Zhang et al., 2023).

### 2.5 Statistical analysis

Statistical analysis was performed by using SPSS (version 27.0, SPSS Inc., Chicago, IL, USA) and PLINK (version 1.9, Shaun Purcell, Boston, MA, USA) software. Continuous variables were expressed as mean ± SD or interquartile range, and categorical variables were expressed as ratios or proportions. Hardy-Weinberg equilibrium was evaluated by PLINK (P ≥ 0.05 was acceptable). Independent samples t-test or Mann-Whitney U test was conducted for the continuous variables. Pearson’s Chi-squared test was performed on categorical variables. Using PLINK, univariate logistic regression analysis was performed for different toxicity, and Bonferroni single-step adjustment was performed for P-values. According to the dominant and recessive model results of PLINK, the genotypes with potential influence on HDMTX-induced toxicities were screened. All variables with a p-value <0.05 in the univariate analysis were included in the binary logistic regression analysis. Odds ratio (OR) and 95% confidence interval (CI) were used to estimate the risk of toxicities for each variable. A p-value <0.05 was considered statistically significant.

## 3 Results

### 3.1 Patient characteristics

The present study included a total of 713 patients (400 male, 313 female) with 3,021 treatment courses were included. Table 2 presents the clinical characteristics of enrolled patients. The age of 713 patients was 55.43 ± 14.32 years. MTX dose was 3.38 ± 0.75 g/m^2^. AUC_0–24h_ (h μmol/L) was 1083.25 ± 323.53 h μmol/L, AUC_0–48h_ was 1105.87 ± 364.80 h μmol/L. Table 3 presents the number of courses with grade 1, 2, 3, and 4 of each toxicity.

**TABLE 2:** Clinical characteristics of 713 patients with PCNSL receiving 3021 HDMTX courses.

Item	Mean ± SD (Min-Max) or numbers (percentages)
Demographic data (713 patients)	
Gender	
Male	400 (56.1%)
Female	313 (43.9%)
Age (year)	55.43 ± 14.32 (18.12–84.24)
Height (cm)	167.06 ± 7.90 (146–192)
Weight (kg)	67.89 ± 11.80 (30–115)
Laboratory test (3021 HDMTX courses)	
ALB (g/L)	37.63 ± 3.31 (24.92–45.75)
Drug exposure (3021 HDMTX courses)	
AUC_0–24h_ (h μmol/L)	1083.25 ± 323.53 (85.25–4351.90)
AUC_0–48h_ (h μmol/L)	1105.87 ± 364.80 (86.03–6708.50)
Co-medicated drugs (3021 HDMTX courses)	
Omeprazole	1623 (53.72)
Ilaprazole	260 (8.61)
PPIs	1797 (59.48)
NSAIDs	130 (4.30)
Furosemide	644 (21.32)
Torasemide	576 (19.07)
Bumetanide	239 (7.91)
Hydrochlorothiazide	45 (1.49)
Levetiracetam	588 (19.46)

NSAIDs, include ibuprofen, acetaminophen, aspirin, and lysine acetylsalicylate; PPIs, include omeprazole, ilaprazole, rabeprazole, pantoprazole, and lansoprazole.

**TABLE 3:** Number of courses per grade of toxicities (n = 3,021).

Toxicity	Grade n (%)				
	0	1	2	3	4
Hepatotoxicity	1866 (61.77)	1072 (35.48)	52 (1.72)	29 (0.96)	2 (0.07)
Nephrotoxicity	2626 (86.92)	337 (11.16)	49 (1.62)	8 (0.26)	1 (0.03)
Hematotoxicity					
Anemia	715 (23.67)	1891 (62.60)	362 (11.98)	53 (1.75)	0 (0)
Neutropenia	2767 (91.59)	118 (3.91)	96 (3.18)	30 (0.99)	10 (0.33)
Leukopenia	2667 (88.28)	169 (5.59)	143 (4.73)	37 (1.22)	5 (0.17)
Thrombocytopenia	2636 (87.26)	312 (10.33)	51 (1.69)	14 (0.46)	8 (0.26)

### 3.2 Association between polymorphisms and HDMTX-related toxicities

Supplementary Table S1 shows the detailed information of selected SNPs. Rs1051226, rs10760502 and rs2273697 were excluded due to high genotype missing rate (>5%). Rs28364006 appeared to be monomorphic in the sample population and was excluded. Rs3758149, rs2413775, and rs11045879 were not consistent with the Hardy-Weinberg equilibrium (p < 0.05).

The association between SNPs and toxicities was analyzed by logistic regression using the dominant and recessive models in PLINK. After Bonferroni single-step adjustment, rs1801131 G allele (p-dom = 0.0005388, p-rec = 1) and rs2236225 A allele (p-dom = 0.02953, p-rec = 1) were associated with a higher risk of hepatotoxicity. Rs1801394 G allele (p-dom = 0.002376, p-rec = 1), rs1801133 G allele (p-dom = 0.04485, p-rec = 1), rs9344 G allele (p-dom = 0.009066, p-rec = 0.1539), and rs2231142 T allele (p-dom = 0.04684, p-rec = 1) were associated with a higher risk of AKI, meanwhile rs1695 A allele (p-dom = 1, p-rec = 2.069e-05) were linked to a lower risk of AKI. Rs2236225 G allele (p-dom = 1, p-rec = 0.006336) and rs2231142 G allele (p-dom = 0.5948, p-rec = 0.002228) were associated with low anemia risk, while rs1045642 A allele (p-dom = 0.04169, p-rec = 1), rs1128503 G allele (p-dom = 0.02983, p-rec = 1), and rs1801131 G allele (p-dom = 0.03646, p-rec = 1) were related to high risk of anemia. Rs2231142 T allele (p-dom = 0.01398, p-rec = 1) and rs717620 T allele (p-dom = 1.309e-05, p-rec = 1) were associated with thrombocytopenia, whereas rs1045642 G allele (p-dom = 1, p-rec = 0.02048), rs1801133 A allele (p-dom = 1, p-rec = 0.0478) showed opposite effect on PLT count. Additionally, rs2236225 A allele (p-dom = 0.01721, p-rec = 1) was found relating to high risk of leukopenia (Table 4).

**TABLE 4:** Association between selected SNPs and HDMTX-related toxicities in 3,021 courses.

SNP	Model	P-BONF					
		Leukopenia	Anemia	Neutropenia	Thrombocytopenia	Hepatotoxicity	Nephrotoxicity
rs10106	DOM	1	1	1	1	1	1
	REC	0.6349	1	1	1	1	1
rs1045642	DOM	1	0.04169	1	1	0.2518	1
	REC	1	1	1	0.02048	1	0.2111
rs1128503	DOM	1	0.02983	1	1	0.2417	1
	REC	1	1	1	1	1	1
rs12995526	DOM	1	1	1	1	1	1
	REC	1	0.4125	1	1	1	1
rs151264360	DOM	1	1	1	1	1	0.1478
	REC	1	1	1	1	1	0.07636
rs1695	DOM	1	0.1184	1	0.1276	0.1638	1
	REC	1	1	1	1	0.2981	2.07E-05
rs1799983	DOM	1	1	0.3274	1	1	1
	REC	1	1	1	1	1	1
rs1801131	DOM	1	0.03646	1	1	0.0005388	1
	REC	1	1	1	1	1	1
rs1801133	DOM	1	1	1	1	1	0.04485
	REC	1	1	1	0.0478	0.7521	1
rs1801394	DOM	1	1	1	1	1	0.002376
	REC	1	1	1	1	1	1
rs1805087	DOM	1	1	1	1	1	1
	REC	1	1	1	1	1	1
rs2231142	DOM	0.8021	0.5948	1	0.01398	1	0.4684
	REC	1	0.002228	1	1	1	1
rs2236225	DOM	0.01721	1	0.1502	1	0.02953	1
	REC	1	0.006336	1	1	1	1
rs2274407	DOM	1	1	1	1	1	1
	REC	1	1	1	1	1	1
rs2274976	DOM	1	1	1	1	0.7987	1
	REC	1	0.08484	1	1	1	1
rs2298383	DOM	1	1	1	1	1	1
	REC	1	1	1	1	0.1215	1
rs2372536	DOM	0.7643	0.4966	1	1	1	1
	REC	1	0.8312	1	1	1	1
rs3740065	DOM	1	1	1	1	1	1
	REC	1	1	1	0.488	1	1
rs4149081	DOM	1	0.9418	1	1	1	1
	REC	1	1	1	1	1	1
rs4673993	DOM	0.6648	1	1	1	1	1
	REC	1	1	1	1	1	1
rs717620	DOM	1	1	1	1.31E-05	1	1
	REC	1	1	1	1	1	1
rs7563206	DOM	1	1	1	1	1	1
	REC	1	0.1746	1	1	1	1
rs9344	DOM	1	0.07231	1	0.3112	1	0.009066
	REC	1	1	1	1	1	0.1539

### 3.3 Population pharmacokinetic model

The referenced population pharmacokinetic model was characterized using a three-compartment model in conjunction with a proportional residual model. Covariate effects on model parameters were evaluated using forward addition and backward elimination approaches. The estimated glomerular filtration rate (eGFR), blood urea nitrogen (BUN), ALT, and a combined genotype of ABCC-ABCG-ADORA2A were identified as significant covariates impacting the clearance (CL) of MTX. Additionally, total protein (TP) was found to be a significant covariate influencing inter-compartmental clearance (Q). The results in the model development procedure were in Supplementary Table S2. Detailed parameter estimates and Bootstrap results of model were in Supplementary Table S3. Goodness-of-fit plots for the base and final models (Supplementary Figure S1) show strong correlations between observed and predicted concentrations. CWRES plots indicate that most residuals fall within two standard deviations and are evenly distributed, with no significant biases observed. The VPC analysis (Supplementary Figure S2) demonstrated that most observed concentrations fell within the model’s 80% prediction intervals, with median observed concentrations closely matching the predicted values, indicating acceptable predictive performance (Wei et al., 2025).

### 3.4 Univariate and multivariate analysis of risk factors for HDMTX-related toxicities

In our patient population, there are relatively few courses in which grades 2–4 occur. Statistically, the results of a separate analysis in each grade are not particularly meaningful. Therefore, we divided the 3,021 courses into two types for univariate and multifactorial analysis: with toxic events and without toxic events. Univariate analyses were used to test the relationship between the patient characteristics and toxicities. The selected patient characteristics were gender, age, body weight, plasma ALB level, AUC_0–24h_, AUC_0–48h_, and co-medications (including omeprazole, ilaprazole, rabeprazole, pantoprazole, lansoprazole, ibuprofen, acetaminophen, aspirin, lysine acetylsalicylate, furosemide, torasemide, bumetanide, hydrochlorothiazide, and levetiracetam).

In general, SNPs with potential influence (p < 0.05 with Bonferroni adjustment, detailed in Table 4) and clinical characteristics with p < 0.05 in univariate analyses were stepwise entered into a multivariate binary logistic regression model to identify predictive factors for occurrence of toxicities. The outcome of predictive factors for HDMTX-induced toxicities in patients with PCNSL is shown in Tables 5–10.

**TABLE 5:** Multivariate analysis for HDMTX-related anemia in 3021 HDMTX courses.

Factor	Type	Anemia		
		OR	95% CI	P
Gender	Male	reference	-	-
	Female	0.199	0.159–0.249	0
AUC_0–24h_	-	0.989	0.984–0.995	0
AUC_0–48h_	-	1.01	1.004–1.015	0.001
ALB	-	0.803	0.776–0.831	0
Omeprazole	-	0.753	0.426–1.329	0.328
Ilaprazole	-	1.507	0.92–2.468	0.104
PPIs	-	0.998	0.54–1.845	0.995
NSAIDs	-	1.228	0.702–2.148	0.471
Furosemide	-	2.434	1.806–3.279	0
Torasemide	-	2.985	2.191–4.066	0
Bumetanide	-	3.775	2.306–6.178	0
Levetiracetam	-	1.46	1.106–1.928	0.008
rs1045642	GG	reference	-	-
	AA + AG	0.739	0.591–0.924	0.008
rs1128503	AA	reference	-	-
	GG + AG	1.459	1.175–1.812	0.001
rs1801131	TT	reference	-	-
	GG + GT	0.763	0.617–0.945	0.013
rs2231142	GG + GT	reference	-	-
	TT	0.751	0.536–1.053	0.097
rs2236225	GG + AG	reference	-	-
	AA	0.568	0.382–0.844	0.005

As shown in Table 5, the risk factors for the occurrence of anemia were as follows: higher AUC_0–48h_ [OR = 1.01, p = 0.001], co-medications (furosemide [OR = 2.434, p = 0], torasemide [OR = 2.985, p = 0], bumetanide [OR = 3.775, p = 0], and levetiracetam [OR = 1.46, p = 0.008]), and genotype GG + AG of rs1128503 [OR = 1.459, p = 0.001]. Protective factors for anemia were female [OR = 0.199, p = 0], higher AUC_0–24h_ [OR = 0.989, p = 0], higher level of ALB [OR = 0.803, p = 0], genotype AA + AG of rs1045642 [OR = 0.739, p = 0.008], genotype GG + GT of rs1801131 [OR = 0.763, p = 0.013], and genotype AA of rs2236225 [OR = 0.568, p = 0.005].

For neutropenia (Table 6), co-use with torasemide [OR = 1.724, p = 0] was presented as a risk factor, while older age [OR = 0.986, p = 0.003] was associated with lower risk. For leucopenia (Table 7), multivariate analysis showed that AUC_0–48h_ [OR = 1.006, p = 0], combination of torasemide [OR = 1.383, p = 0.024, and genotype AA + AG of rs2236225 [OR = 1.458, p = 0.001] were related to higher risk. High ALB level [OR = 0.943, p = 0.001] and AUC_0–24h_ [OR = 0.993, p = 0] were protective factors.

**TABLE 6:** Multivariate analysis for HDMTX-related neutropenia in 3021 HDMTX courses.

Factor	Neutropenia		
	OR	95% CI	P
Age	0.986	0.976–0.995	0.003
PPIs	0.984	0.75–1.291	0.909
Torasemide	1.724	1.278–2.326	0

**TABLE 7:** Multivariate analysis for HDMTX-related leukopenia in 3021 HDMTX courses.

Factor	Type	Leukopenia		
		OR	95% CI	P
Gender	Male	reference	-	-
	Female	1.195	0.928–1.54	0.168
Weight	-	0.999	0.987–1.011	0.822
AUC_0–24h_	-	0.993	0.991–0.996	0
AUC_0–48h_	-	1.006	1.003–1.008	0
ALB	-	0.943	0.911–0.976	0.001
Omeprazole	-	0.581	0.261–1.294	0.184
Ilaprazole	-	0.486	0.232–1.02	0.056
PPIs	-	2.066	0.899–4.747	0.087
Torasemide	-	1.383	1.044–1.83	0.024
rs2236225	GG	reference	-	-
	AA + AG	1.458	1.16–1.832	0.001

Thrombocytopenia was linked to female [OR = 1.519, p = 0], co-meditating with furosemide [OR = 1.485, p = 0.003], GG type of rs1801133 [OR = 1.379, p = 0.011], TT + GT type of rs2231142 [OR = 1.355, p = 0.009], and TT + CT type of rs717620 [OR = 1.661, p = 0], while older patients [OR = 0.969, p = 0], higher ALB [OR = 0.894, p = 0], and AA genotype of rs1045642 [OR = 0.534, p = 0.001] showed protective effects (Table 8).

**TABLE 8:** Multivariate analysis for HDMTX-related thrombocytopenia in 3021 HDMTX courses.

Factor	Type	Thrombocytopenia		
		OR	95% CI	P
Gender	Male	reference	-	-
	Female	1.519	1.201–1.921	0
Age	-	0.969	0.96–0.977	0
ALB	-	0.894	0.863–0.927	0
Omeprazole	-	1.807	0.76–4.298	0.181
Ilaprazole	-	0.749	0.388–1.445	0.389
PPIs	-	0.806	0.329–1.977	0.638
Furosemide	-	1.485	1.142–1.932	0.003
rs1045642	AG + GG	reference	-	-
	AA	0.534	0.373–0.765	0.001
rs1801133	AA + AG	reference	-	-
	GG	1.379	1.078–1.764	0.011
rs2231142	GG	reference	-	-
	TT + GT	1.355	1.078–1.702	0.009
rs717620	CC	reference	-	-
	TT + CT	1.661	1.326–2.081	0

As for hepatotoxicity (Table 9), the older age [OR = 0.985, p = 0], AUC_0–48h_ [OR = 0.999, p = 0.07], use of ilaprazole [OR = 0.451, p = 0.001] and furosemide [OR = 0.775, p = 0.012] have less risk, female patients [OR = 1.393, p = 0], GG and GT types of rs1801131 [OR = 1.254, p = 0.007] compared with its homozygote genotypes TT were associated with hepatotoxicity.

**TABLE 9:** Multivariate analysis for HDMTX-related hepatotoxicity in 3021 HDMTX courses.

Factor	Type	Hepatotoxicity		
		OR	95% CI	P
Gender	Male	reference	-	-
	Female	1.393	1.189–1.631	0
Age	-	0.985	0.979–0.991	0
AUC_0–24h_	-	1.002	1.001–1.004	0.007
AUC_0–48h_	-	0.999	0.997–1	0.07
Omeprazole	-	0.979	0.556–1.722	0.941
Ilaprazole	-	0.451	0.28–0.728	0.001
PPIs	-	0.649	0.361–1.165	0.147
Furosemide	-	0.775	0.636–0.945	0.012
rs1801131	TT	reference	-	-
	GG + GT	1.254	1.063–1.479	0.007

Table 10 illustrates patients with higher body weight [OR = 1.02, p = 0.002], higher AUC_0–48h_ [OR = 1.022, p = 0], GG type of rs1695 [OR = 1.385, p = 0.013], and GG type of rs1801133 [OR = 1.3423, p = 0.041] present higher risk of nephrotoxicity. Female associated with less risk compared with male [OR = 0.138, p = 0]. Factors like AUC_0–24h_ [OR = 0.979, p = 0] and genotype GG and AG of rs1801394 [OR = 0.614, p = 0] also linked to less risk.

**TABLE 10:** Multivariate analysis for HDMTX-related nephrotoxicity in 3021 HDMTX courses.

Factor	Type	Nephrotoxicity		
		OR	95% CI	P
Gender	Male	reference	-	-
	Female	0.138	0.095–0.2	0
Weight	-	1.02	1.007–1.032	0.002
AUC_0–24h_	-	0.979	0.974–0.984	0
AUC_0–48h_	-	1.022	1.016–1.027	0
ALB	-	0.969	0.933–1.006	0.095
Omeprazole	-	0.872	0.432–1.759	0.701
Ilaprazole	-	1.263	0.706–2.257	0.431
PPIs	-	1.27	0.599–2.692	0.533
rs1695	AA + AG	reference	-	-
	GG	1.385	1.07–1.793	0.013
rs1801133	AA + AG	reference	-	-
	GG	1.342	1.012–1.778	0.041
rs1801394	AA	reference	-	-
	GG + AG	0.614	0.477–0.79	0
rs2231142	GG	reference	-	-
	TT + GT	1.182	0.921–1.517	0.189
rs9344	AA	reference	-	-
	GG + AG	0.856	0.659–1.112	0.244

## 4 Discussion

### 4.1 Drug exposure

During the treatment, delayed MTX excretion in patients leads to increased drug exposure, which subsequently raises the incidence of adverse events. Utilizing drug exposure as a reference index to guide PCNSL treatment can provide early warnings before toxicity occurs, allowing for timely dose adjustments and leucovorin rescue protocols. Studies have found that the AUC of MTX (0–96 h) may be correlate with both toxicity and efficacy (Joerger et al., 2010; Joerger et al., 2012). In a retrospective IELSG study on PCNSL, AUC (>1100 h μmol/L) was identified as an independent predictor of PCNSL treatment efficacy (Ferreri et al., 2004). Another study involving 55 PCNSL patients indicated that for every 100 h μmol/L increase in AUC, disease progression reduced by 18% and overall survival improved by 27%, but no significant relationship was observed between AUC and MTX toxicity (Joerger et al., 2010). To explore the specific impact of AUC on toxicity, we used a population pharmacokinetic model to fit relatively stable drug-time curves for each treatment cycle.

The model used a nonlinear mixed-effects model with a two-compartment model with first-order elimination to describe the pharmacokinetic process of MTX in patients. An exponential error model was used to assess inter-individual variability between parameters, and a proportional model was used to describe residual variability between MTX concentrations. Covariates included in the model were eGFR, ALT, TP, and concomitant use of omeprazole. Scatter plots were used to assess the fit of the base and final models, and typical parameter estimates, standard errors, and 95% CIs obtained from the original data set were consistent with bootstrap results, indicating good stability and reproducibility of the final model. Most of the actual concentrations in the VPC assay fell within the 90% prediction interval of the model, indicating that the predictive ability of the model was adequately predictive.

Our analysis showed that AUC_0–24h_ and AUC_0–48h_ serve as predictive and risk factors respectively for anemia, leukopenia, and nephrotoxicity during the treatment cycles. Patients with larger AUC_0–24h_ had a higher risk of hepatotoxicity, and higher AUC_0–48h_ was associated with lower risk. However, it cannot be ignored that although the results are statistically significant, the ORs related to AUC are very close to 1.

MTX and its metabolite 7-OH MTX can form pH-dependent crystalline precipitates in the renal tubule lumen, leading to crystal nephropathy and renal damage (Howard et al., 2016). MTX is primarily excreted through the kidneys, and impaired renal function results in prolonged exposure to high drug concentrations. Meanwhile, MTX continuously inhibits dihydrofolate reductase, targeting DNA synthesis and suppressing the growth and division of hematopoietic cells in the bone marrow (Bedoui et al., 2019; Hamed et al., 2022). Hence, hematotoxicity and nephrotoxicity were consistent in the results. Apart from affecting folate metabolism, MTX-polyglutamates can accumulate in liver cells and lead to plasma membrane lysis, releasing a large number of reactive oxygen species, causing oxidative damage (Li et al., 2024). However, none of these can explain our results well, and the specific effect of AUC on toxicity is rarely studied.

### 4.2 Plasma ALB

Retrospective studies of hematologic malignancies patients undergoing HDMTX therapy indicate low ALB levels are significantly associated with delayed MTX clearance (Li et al., 2024). Amitai I et al. found that an ALB level <3.6 g/dL (OR = 4.17, 95% CI: 1.04–6.5, p = 0.04) is strongly associated with AKI (Amitai et al., 2020). This correlation was also observed by Khera S et al., where lower ALB levels (<35 g/L) were an independent risk factor for MTX-induced nephrotoxicity in pediatric ALL patients (OR = 4.71, 95% CI: 1.06–9.86, p = 0.04) (Khera et al., 2022). Higher ALB levels were significantly associated with a lower incidence of hematotoxicity in our study. Methotrexate has a protein binding rate of approximately 50%, and albumin accounts for about 80% of intravascular oncotic pressure. Low serum albumin levels facilitate fluid shift into the interstitial space, leading to extravascular fluid accumulation (such as ascites, pleural effusion, and intracranial effusion). This promotes the diffusion of the polar molecule MTX into third-space fluids, prolonging drug exposure time (Li et al., 2024; Reiss et al., 2016). Additionally, hypoalbuminemia reduces intravascular volume and decreases renal perfusion, which directly contributes to renal injury (Van der Beek et al., 2019). However, the study by Li X et al. in ALL children contradicts our findings, showing higher ALB as a risk factor for MTX-induced leucopenia (OR = 1.084, 95% CI: 1.003–1.171, p = 0.041) and neutropenia (OR = 1.101, 95% CI: 1.019–1.189, p = 0.0015), the odds ratios were very close to 1, suggesting a relatively weak association that may lack clinical significance (Li et al., 2019). Differences in patient characteristics, measurement timing, and adjustment for confounders may have contributed to the observed discrepancy. Further studies are needed to validate these findings across populations.

### 4.3 Gene polymorphisms

After entering cells, MTX can be converted to the polyglutamate form to inhibit dihydrofolate reductase and prevent the conversion of dihydrofolate to tetrahydrofolate (THF) (Xu et al., 2022). In the purine and pyrimidine synthesis pathway, THF is an essential coenzyme in several transmethylation reactions and is also required for tumor cells to synthesize DNA and RNA. Methylenetetrahydrofolate dehydrogenase 1 (MTHFD1) catalyzes the reduction of THF to produce a variety of active folic acid forms (e.g., 5-CH_3_-THF, 10-CH_3_-THF and 5,10-CH_2_-THF) to function as donors of single-carbon units in DNA synthesis (Giletti and Esperon, 2018). In our study, AA mutant homozygote on MTHFD1 rs2236225 was associated with a lower risk of anemia, while the wild-type homozygote GG associated with a lower risk of leukopenia. Studies have shown that A allele can alter the structure of MTHFD domain, reduce the activity of the enzyme, tilt the balance of folic acid, which is conducive to DNA repair and cell proliferation (Erculj et al., 2012). So far, several studies have investigated the effect of MTHFD polymorphism on HDMTX-related toxicity, but conflicting results have been reported and more evidence is needed (Erculj et al., 2012; Windsor et al., 2012; Erculj et al., 2014).

Methylenetetrahydrofolate reductase (MTHFR) can irreversibly convert 5,10-CH_2_-THF to 5-CH_3_-THF (Xu et al., 2022). Rs1801133 and rs1801131 mutations reduce the activity of MTHFR, which is not conducive to protein synthesis, but also block the synthesis of THF, affecting DNA and RNA synthesis (Giletti and Esperon, 2018). In our analysis, the GG genotype in rs1801133 was associated with increased nephrotoxicity and thrombocytopenia, G mutation of rs1801131 is a risk factor for hepatotoxicity and a protective factor for anemia. It is worth mentioning that the influence of rs1801131 is much weaker than that of rs1801133 in reports, and some point out that rs1801131 has no obvious correlation with toxicity (Li et al., 2024).

Methionine synthase converts homocysteine into methionine for protein synthesis, and converts 5-CH_3_-THF into THF (Zhang et al., 2023). Only a few studies have pointed out the relationship between rs1801394 and mucositis with inconsistent results, but we found rs1801394 G allele is reflected as protective factor in occurrence of renal injury (Huang et al., 2008; Gong Y. et al., 2021).

ATP-binding cassette family transporters (involving proteins such as ABCB1, ABCG2 and ABCC1-5) promote the efflux of MTX and its metabolites from cells (Song et al., 2021; Vlaming et al., 2009). Our study found patients with T allele on ABCC2 rs717620 had a higher risk of thrombocytopenia. Liu Y′s study also mentioned T allele at rs717620 showed higher MTX plasma concentration than wild-type CC (Liu et al., 2014). The mutation on rs717620 is located in the 5′-untranslated region and has a negative effect on the activity of ABCC2 promoter. This polymorphism is associated with lower mRNA levels, so the synthesis of ABCC2 is reduced (Grzelj et al., 2021a). This results in diminished MTX elimination and increased *in vivo* exposure, increasing adverse reactions (Grzelj et al., 2021b; Razali et al., 2020).

In our study, at ABCB1 rs1045642, compared with the CC genotype, patients with the T allele had a lower risk of anemia and thrombocytopenia, which is consistent with the results of Jian Han’s study (Han et al., 2021). Gregers’ study also showed that patients with TT and CT variants had better liver function (Gregers et al., 2015). In the pharmacokinetic model established by Kim et al., patients with T allele had a lower MTX clearance rate, meaning higher drug exposure (Kim et al., 2012). Our study found that the G allele at rs1128503 is a risk factor for anemia. In Zaruma-Torres’s study, the mutation of ABCB1 rs1128503 was not related to the common adverse reactions of MTX, but showed a protective effect on bone marrow suppression (Zaruma-Torres et al., 2015). As the product of ABCB1 gene, P-gp is highly expressed in tumor cells (Li et al., 2023). The synonymous SNP, ABCB1 rs1128503 and rs1045642 polymorphisms did not change the amino acid sequence in the protein it encodes, but may be associated with decreased P-gp expression, and P-gp-mediated MTX transport may be affected (de Rotte et al., 2012).

The protein encoded by the ABCG2 gene not only affects the efflux of MTX, but also linked to anticancer drug resistance (Wang et al., 2011; Gorczyca and Aleksunes, 2020). In our patient population, A allele on rs2231142 have a higher risk of thrombocytopenia. The study by Esmaili MA et al. supported that ABCB1 rs1045642 CT genotype and ABCG2 rs2231142 AC genotype had significantly higher plasma MTX levels within 48 h, with no significant association with MTX-related hematopoietic and hepatic toxicities (Esmaili et al., 2020). The mutant allele A on ABCG2 rs2231142 is related to the decreased function of MTX transporter, leading to higher accumulation of MTX-PG in cells and an increased possibility of toxicity (Li et al., 2023; Baghdadi et al., 2018).

Patients with GG genotype at Glutathione S-transferase Pi 1 (GSTP1) rs1695 may be more prone to nephrotoxicity when treated with HDMTX. The association between GG genotype and central nervous system toxicity was also observed by Kishi S et al. (Kishi et al., 2007) GSTP is a phase II metabolic enzyme, which reduces the toxic of chemotherapy by catalyzing the combination of electrophilic substances and glutathione (Gong J. Y. et al., 2021). In addition to its role in detoxification, GSTP1 is involved in the activation of peroxiredoxin 6 (PRDX6). PRDX6 is a bifunctional antioxidant enzyme that protects cells from oxidative damage by reducing hydrogen peroxide and lipid peroxide (do Nascimento et al., 2021; de Sousa Barros et al., 2022). Reduced enzyme activity was observed in G variants, which not only prevents cellular detoxification, but may also affect the antioxidant response (Baghaei et al., 2022).

### 4.4 Co-medication

Loop diuretics exert diuretic effects by inhibiting Na^+^/K^+^/2Cl^-^ cotransporters in the thick ascending limb of the renal loop. On one side, the potent diuretic effect of diuretics may accelerate the excretion of MTX together with its metabolites, and reduce the retention of MTX. However, furosemide, torasemide and bumetanide require renal excretion, which will compete with MTX for OAT transporters secreted by renal tubules, resulting in decreased MTX excretion (Nigam et al., 2015; Nigam, 2018). And from a general dimension, the discharge of a large amount of urine reduces the effective circulating blood volume, leading to renal hypoperfusion, decreased glomerular filtration rate, and decreased MTX excretion (Howard et al., 2016). In our study, the combination of furosemide, torasemide and bumetanide with MTX was significantly associated with the occurrence of hematotoxicity, whereas furosemide appeared to have a decrease in hepatotoxicity. There are other scholars pointed out that furosemide is positively correlated with the occurrence of nephrotoxicity (Liang et al., 2023).

Levetiracetam is observed as a risk factor for methotrexate-induced anemia in our study. There were two reports of cases of delayed MTX elimination while taking levetiracetam concurrently (Bain et al., 2014; Parentelli et al., 2013). However, some studies showed no drug interaction between levetiracetam and HDMTX (DeFino et al., 2021; Lou et al., 2020; Reeves et al., 2016). Both levetiracetam and MTX are primarily excreted through kidney. Co-administration may compete for secretion from the renal tubules, leading to blocked MTX excretion (Reeves et al., 2016).

### 4.5 Age

Existing studies have explained that in adult patients, delayed excretion of MTX is more pronounced with age, and is a risk factor for acute hepatotoxicity (Zang et al., 2019; Wang et al., 2023). In contrast, age was shown to be a protective factor for hepatotoxicity and hematotoxicity in our logistic regression, with OR ranging from 0.98 to 1, indicating that the influence was weak and age is not a strong predictor of toxicity in the our data set. The observed discrepancy in age may be due to the different doses administered to patients with different ages. We first conducted a linear analysis between patient age and drug dosage, which yielded an R^2^ value of 0.0092, indicating a very weak correlation. Therefore, we further stratified patients into two groups for subgroup analysis: ≤ 60 years, >60 years. The difference in dose/m^2^ between the two groups was compared. We found that older patients received lower doses during treatment cycles (p = 0.03). This may explain why older patients experienced less toxicity.

### 4.6 Limitations

Our study also has certain limitations: Firstly, the number of treatment cycles with grade 2–4 toxicity is small, especially for grade 3 or 4, so we divided the patients into two groups based on whether they experienced toxicity or not for statistical analysis. Secondly, we only have information on the drug administration during the hospitalization period, and PCNSL patients in our hospital are usually treated with combination chemotherapy, so the interference of other chemotherapy drugs cannot be excluded. Thirdly, as we did not analyze the relationship between 7-OH MTX and toxicity, the effect of 7-OH MTX on hepatotoxicity is unknown (Bremnes et al., 1991; Fuskevag et al., 2000). Given the rarity of PCNSL, the available patient population is inherently limited. This study included all eligible patients from our institution between 2016 and February 2024. And the data cleaning procedures reduced the final sample size, future studies with larger, multicenter cohorts are warranted to validate and extend these findings. We acknowledge that patient comorbidities and concurrent treatments may influence toxicity outcomes. However, due to limitations in the completeness and consistency of retrospective clinical records, we were unable to comprehensively adjust for these confounders. In our study, loop diuretics were associated with increased nephrotoxicity, possibly due to competition for renal tubular secretion with methotrexate. However, due to the lack of MTX clearance data, this mechanism could not be verified directly. After reading some case reports and references, the observed association between levetiracetam and hematologic toxicity also lacks established pharmacologic support. Additionally, diuretics are commonly prescribed for clinical conditions such as heart failure, edema, or impaired renal function—factors which may themselves predispose patients to toxicity. These underlying indications were not fully captured in our dataset, and the possibility of confounding cannot be excluded. These findings should be interpreted with caution, and further studies are needed to explore the underlying mechanisms. One methodological limitation of this study is that formal multicollinearity diagnostics were not conducted for the logistic regression models. Although efforts were made to minimize potential redundancy among covariates by selecting clinically relevant variables and avoiding highly correlated predictors, the lack of quantitative assessment of multicollinearity may affect the interpretability of results.

## 5 Conclusion

The results show that in PCNSL Chinese patients, higher plasma ALB was associated with a lower risk of HDMTX-related toxicities; the combination of loop diuretics and levetiracetam might increase the occurrence of toxic reactions. MTHFR (rs1801133) GG, ABCB1 (rs1128503) GG and AG, ABCG2 (rs2231142) AA and AC, and ABCC2 (rs717620) TT and GT genotypes were associated with increased MTX toxicities, whereas rs1045642 TT and CT, rs1801394 GG and AG genotypes might have protective effects.

## Data Availability

The original contributions presented in the study are included in the article/Supplementary Material, further inquiries can be directed to the corresponding authors.
