# Supplementary material for: Risk factors associated with high-dose methotrexate induced toxicities in primary central nervous system lymphoma
Source: Front Pharmacol. 2025 Aug 4;16:1561818. doi: 10.3389/fphar.2025.1561818 (PMC12358356; doi:10.3389/fphar.2025.1561818)
Supplement: Supplementary file 1 [file DataSheet1.docx]

**TITLE**

Risk factors associated with high-dose methotrexate induced toxicities in primary central nervous system lymphoma

**JOURNAL NAME**

Frontiers in Pharmacology

**AUTHORS**

Wenshu Li^1,2^†, Sitian Zhang^3^†, Ruoyun Wu^1,2^†, Ying Li^4^, Shifeng Wei^1,2^, Lin Fu^4^, Xuefei Sun^4^, Yuanbo Liu^4*^, Zhigang Zhao^1,2*^, Shenghui Mei^1,2*^

†These authors contributed equally to this work and should be considered co-first authors.

**AUTHORS’ AFFILIATIONS**

1 Department of Pharmacy, Beijing Tiantan Hospital, Capital Medical University, 119 Nansihuan West Road, Fengtai District, Beijing 100070, P. R. China

2 Department of Clinical Pharmacology, College of Pharmaceutical Sciences, Capital Medical University, Beijing 100069, P. R. China

3 School of Basic Medical Sciences, Capital Medical University, Beijing 100069, P. R. China

4 Department of Hematology, Beijing Tiantan Hospital, Capital Medical University, 119 Nansihuan West Road, Fengtai District, Beijing 100070, P. R. China

**CORRESPONDING AUTHOR**

Yuanbo Liu: Department of Hematology, Beijing Tiantan Hospital, Capital Medical University, 119 Nansihuan West Road, Fengtai District, Beijing, 100070, P.R. China; E-mail: [yuanbol@ccmu.edu.cn](mailto:yuanbol@ccmu.edu.cn)

Zhigang Zhao: Department of Pharmacy, Beijing Tiantan Hospital, Capital Medical University, 119 Nansihuan West Road, Fengtai District, Beijing, 100070, P.R. China; E-mail: [1022zzg@sina.com](mailto:1022zzg@sina.com)

Shenghui Mei: Department of Pharmacy, Beijing Tiantan Hospital, Capital Medical University, 119 Nansihuan West Road, Fengtai District, Beijing, 100070, P.R. China; E-mail: [meishenghui1983@126.com](mailto:meishenghui1983@126.com)

**Supplementary Table 1 Information of the 30 selected SNPs and p-values of Hardy Weinberg equilibrium**

| SNP | Gene | SNP position (bp) | Minor allele(A1) | Major allele(A2) | Genotype  (A1A1/A1A2/A2A2) | P-hwe |
| --- | --- | --- | --- | --- | --- | --- |
|  |  |  |  |  |  |  |
| rs10106 | FPGS | Chr9: 127,813,796 | T | C | 89/327/295 | 0.9349 |
| rs1045642 | ABCB1 | Chr7: 87,509,329 | A | G | 109/330/265 | 0.6927 |
| rs1128503 | ABCB1 | Chr7: 87,550,285 | G | A | 89/323/299 | 0.9345 |
| rs12995526 | ATIC | Chr2: 215,333,248 | T | C | 41/258/411 | 0.9183 |
| rs151264360 | TYMS | Chr18: 673,444 - 673,452 | I | D | 76/321/312 | 0.673 |
| rs1695 | GSTP1 | Chr11: 67,585,218 | G | A | 22/224/465 | 0.4643 |
| rs1799983 | NOS3 | Chr7: 150,999,023 | T | G | 10/119/582 | 0.1968 |
| rs1801131 | MTHFR | Chr1: 11,794,419 | G | T | 14/200/496 | 0.2667 |
| rs1801133 | MTHFR | Chr1: 11,796,321 | G | A | 169/337/201 | 0.2289 |
| rs1801394 | MTRR | Chr5: 7,870,860 | G | A | 42/268/389 | 0.6888 |
| rs1805087 | MTR | Chr1: 236,885,200 | G | A | 4/112/597 | 0.8086 |
| rs2231142 | ABCG2 | Chr4: 88,131,171 | T | G | 66/303/341 | 1 |
| rs2236225 | MTHFD1 | Chr14: 64,442,127 | A | G | 36/265/406 | 0.468 |
| rs2274407 | ABCC4 | Chr13: 95,206,781 | A | C | 17/160/534 | 0.2628 |
| rs2274976 | MTHFR | Chr1: 11,790,870 | T | C | 3/72/633 | 0.4637 |
| rs2298383 | ADORA2A | Chr22: 24,429,543 | C | T | 173/360/177 | 0.764 |
| rs2372536 | ATIC | Chr2: 215,325,297 | G | C | 48/268/396 | 0.7679 |
| rs3740065 | ABCC2 | Chr10: 99,845,936 | G | A | 101/318/290 | 0.3738 |
| rs4673993 | ATIC | Chr2: 215,347,616 | C | T | 53/267/385 | 0.4979 |
| rs717620 | ABCC2 | Chr10: 99,782,821 | T | C | 31/249/431 | 0.5833 |
| rs7563206 | ATIC | Chr2: 215,325,931 | T | C | 40/257/413 | 1 |
| rs9344 | CCND1 | Chr11: 69,648,142 | G | A | 153/366/191 | 0.4076 |
| rs4149081 | SLCO1B1 | Chr12: 21,225,087 | A | G | 118/315/280 | 0.06946 |
| rs1051266 | SLC19A1 | Chr21: 45,537,880 | T | C | 136/296/183 | 0.4652 |
| rs10760502 | FPGS | Chr9: 127,802,988 | A | G | 13/38/494 | 9.63E-10 |
| rs11045879 | SLCO1B1 | Chr12: 21,229,685 | C | T | 118/268/296 | 5.35E-05 |
| rs2273697 | ABCC2 | Chr10: 99,804,058 | A | G | 5/106/418 | 0.6608 |
| rs2413775 | SLC28A2 | Chr15: 45,252,090 | A | T | 17/240/452 | 0.02221 |
| rs28364006 | ABCC1 | Chr16: 16,134,392 | 0 | A | 0/0/628 | 1 |
| rs3758149 | GGH | Chr8: 63,039,169 | A | G | 57/164/485 | 5.27E-11 |

**Supplementary Table 2 Results in the population pharmacokinetic model development procedure of the final model**

| **Model No.** | **Model description** | **OFV** | **∆OFV** | ***P* value** |
| --- | --- | --- | --- | --- |
| Forward addition |  |  |  |  |
| 1 | Base model | 17.69 |  |  |
| 2 | Add eGFR on CL in model 1 | –454.20 | –471.89 | < 0.05 |
| 3 | Add TP on Q in model 2 | –511.01 | –56.81 | < 0.05 |
| 4 | Add BUN on CL in model 3 | –536.21 | –25.20 | < 0.05 |
| 5 | Add GEN on CL in model 5 | –564.16 | –9.95 | < 0.05 |
| Backward elimination |  |  |  |  |
| 10 | Remove eGFR on CL in model 6 (Gene-model) | –104.88 | 459.29 | < 0.01 |
| 11 | Remove TP on Q in model 6 (Gene-model) | –514.67 | 49.49 | < 0.01 |
| 12 | Remove BUN on CL in model 6 (Gene-model) | –534.41 | 29.75 | < 0.01 |
| 13 | Remove ALT on CL in model 6 (Gene-model) | –546.25 | 17.91 | < 0.01 |
| 14 | Remove GEN on CL in model 6 (Gene-model) | –554.21 | 9.95 | < 0.01 |

**Supplementary Table 3 Parameter estimates and Bootstrap results of Methotrexate population pharmacokinetic model**

| Parameter | Base model | | Final model | | Bootstrap model | |
| --- | --- | --- | --- | --- | --- | --- |
|  | Estimate (%RSE) | 95% CI | Estimate (%RSE) | 95% CI | Median (%RSE) | 95% CI |
| CL (L/h) | 8.35 (2.76) | (7.9, 8.8) | 8.45 (2.98) | (7.95, 8.94) | 8.50 (6.62) | (7.38, 9.71) |
| Q_1_ (L/h) | 0.05 (7.41) | (0.04, 0.06) | 0.04 (8.14) | (0.03, 0.05) | 0.04 (13.81) | (0.03, 0.05) |
| Q_2_ (L/h) | 0.10 (4.85) | (0.09, 0.11) | 0.09 (5.14) | (0.08, 0.10) | 0.09 (10.36) | (0.07, 0.11) |
| Vc (L) | 33.4 (3.34) | (31.21, 35.58) | 33.29 (3.50) | (31.00, 35.57) | 33.52 (9.15) | (27.7, 40.44) |
| V_P1_ (L) | 24.84 (11.00) | (19.48, 30.2) | 17.85 (11.48) | (13.84, 21.87) | 18.32 (24.57) | (11.77, 30.67) |
| V _P2_ (L) | 1.21 (3.97) | (1.12, 1.31) | 1.14 (4.36) | (1.04, 1.24) | 1.15 (8.94) | (0.96, 1.37) |
| *θ_eGFR_* | - | - | 0.67 (2.10) | (0.64, 0.70) | 0.67 (11.04) | (0.56, 0.85) |
| *Θ_BUN_* | - | - | -0.08 (10.20) | (-0.09, -0.06) | -0.07 (38.35) | (-0.13, -0.02) |
| *θ_ALT_* | - | - | 0.03 (14.23) | (0.02, 0.03) | 0.03 (42.92) | (0.00, 0.05) |
| *θ_TP_* | - | - | -1.72 (8.12) | (-1.99, -1.44) | -1.73 (43.04) | (-3.30, -0.31) |
| *θ_ABCC4-ABCG2-ADORA2A_* | - | - | -0.09 (-30.63) | (-0.14, -0.03) | -0.08 (28.65) | (-0.13, -0.04) |
| IIV_CL_ (CV%) | 31.9 (1.76) | - | 27.1 (1.88) | - | 26.81 (4.04) | - |
| IIV_Q1_ (CV%) | 105.35 (10.70) | - | 99.01 (9.47) | - | 99.92 (14.97) | - |
| IIV_Vc_ (CV%) | 16.25 (5.95) | - | 20.27 (5.91) | - | 21.26 (6.47) | - |
| IIV_Vp1_ (CV%) | 84.73 (21.04) | - | 78.1 (25.46) | - | 79.68 (25.05) | - |
| IIV_Vp2_ (CV%) | 26.91 (5.00) | - | 27.2 (5.23) | - | 27.34 (5.52) | - |
| σ (proportional) | 75.04 (1.00) | (73.57, 76.5) | 73.79 (1.07) | (72.24, 75.34) | 73.75 (3.01) | (69.66, 78.42) |


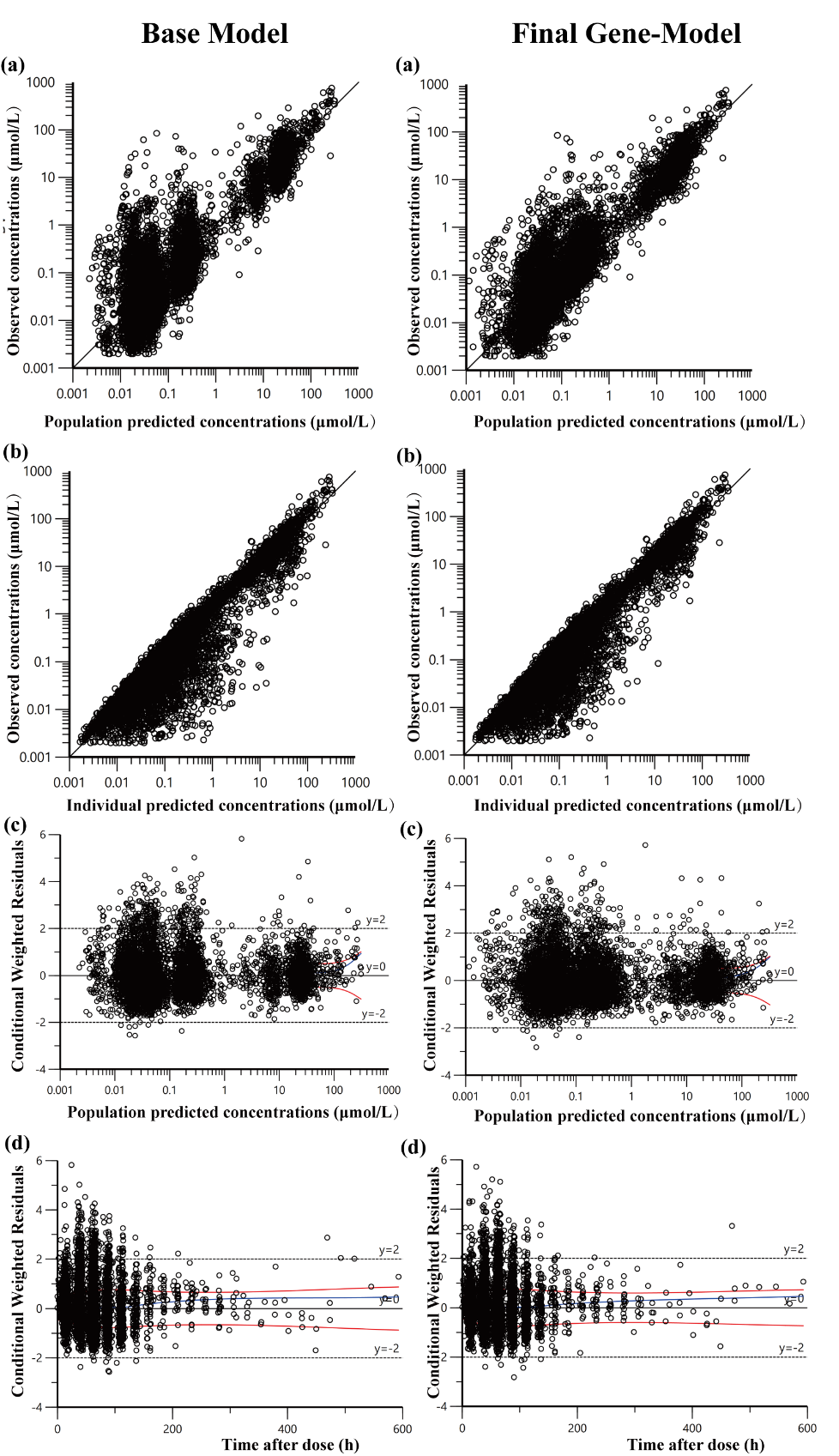
**Supplementary Figure 1 Diagnostic goodness-of fit plots of base model and final model**

(a) observed versus population predicted concentrations (PRED); (a) observed versus individual predicted concentrations (IPRED); (c) conditional weighted residual (CWRES) versus PRED; (d) CWRES versus time after dose (TAD). In plots (c) and (d), the two red lines represent the distribution of absolute CWRES values of the data and its mirror image, respectively.


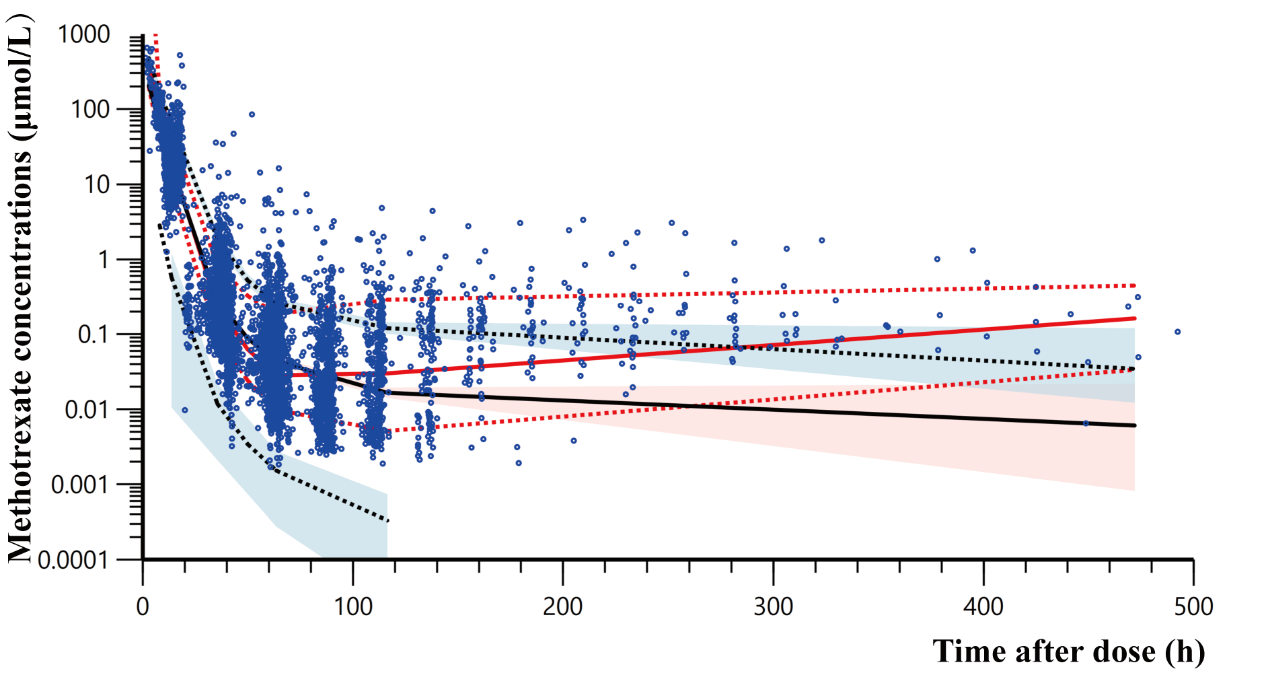
**Supplementary Figure 2 Visual predictive check results of final model.**

The solid black line represents the median predicted concentration, while the two black dashed lines represent the 10th and 90th percentiles of the predicted concentration. The solid red line represents the median observed concentrations, while the two red dashed lines represent the 10th and 90th percentiles of the observed concentrations. The light red and light blue regions respectively represent the 95% confidence intervals for the median, 10th percentile and 90th percentile of the predicted concentrations. The observed data for methotrexate are denoted by blue dots.
